# Proximity control of interlayer exciton-phonon hybridization in van der Waals heterostructures

**DOI:** 10.1038/s41467-021-21780-6

**Published:** 2021-03-19

**Authors:** Philipp Merkl, Chaw-Keong Yong, Marlene Liebich, Isabella Hofmeister, Gunnar Berghäuser, Ermin Malic, Rupert Huber

**Affiliations:** 1 Department of Physics, University of Regensburg, Regensburg, Germany; 2 Department of Physics, Philipps-Universität Marburg, Marburg, Germany; 3 Department of Physics, Chalmers University of Technology, Gothenburg, Sweden

**Keywords:** Two-dimensional materials, Infrared spectroscopy, Two-dimensional materials

## Abstract

Van der Waals stacking has provided unprecedented flexibility in shaping many-body interactions by controlling electronic quantum confinement and orbital overlap. Theory has predicted that also electron-phonon coupling critically influences the quantum ground state of low-dimensional systems. Here we introduce proximity-controlled strong-coupling between Coulomb correlations and lattice dynamics in neighbouring van der Waals materials, creating new electrically neutral hybrid eigenmodes. Specifically, we explore how the internal orbital 1*s*-2*p* transition of Coulomb-bound electron-hole pairs in monolayer tungsten diselenide resonantly hybridizes with lattice vibrations of a polar capping layer of gypsum, giving rise to exciton-phonon mixed eigenmodes, called excitonic Lyman polarons. Tuning orbital exciton resonances across the vibrational resonances, we observe distinct anticrossing and polarons with adjustable exciton and phonon compositions. Such proximity-induced hybridization can be further controlled by quantum designing the spatial wavefunction overlap of excitons and phonons, providing a promising new strategy to engineer novel ground states of two-dimensional systems.

## Abstract (web summary)

Here, the authors demonstrate proximity-controlled strong-coupling between Coulomb correlations and lattice dynamics in neighbouring van der Waals materials (WSe_2_ and a gypsum layer), creating electrically neutral hybrid exciton-phonon eigenmodes called *excitonic Lyman polarons*.

## Introduction

Heterostructures of atomically thin materials provide a unique laboratory to explore novel quantum states of matter^1–11^. By van der Waals stacking, band structures and electronic correlations have been tailored, shaping moiré excitons^1–5^, Mott insulating^1,8–10^, superconducting^7,10^, and (anti-)ferromagnetic states^6–8^. The emergent phase transitions have been widely considered within the framework of strong electron–electron correlations^12–14^. Yet, theoretical studies have emphasized the role of electron–phonon coupling in atomically thin two-dimensional (2D) heterostructures, which can give rise to a quantum many-body ground state featuring Fröhlich polarons, charge-density waves, and Cooper pairs^15–18^. Unlike in bulk media, electronic and lattice dynamics of different materials can be combined by proximity. In particular, coupling between charge carriers and phonons at atomically sharp interfaces of 2D heterostructures are widely considered a main driving force of quantum states not possible in the bulk, such as high-*T*_c_ superconductivity in FeSe monolayer (ML)/SrTiO_3_ heterostructures^11^, enhanced charge-density wave order in NbSe_2_ ML/hBN heterostructures^19^ and anomalous Raman modes at the interface of WSe_2_/hBN heterostructures^20^. However, disentangling competing effects of many-body electron–electron and electron–phonon coupling embedded at the atomic interface of 2D heterostructures is extremely challenging and calls for techniques that are simultaneously sensitive to the dynamics of lattice and electronic degrees of freedom.

**Fig. 1:**
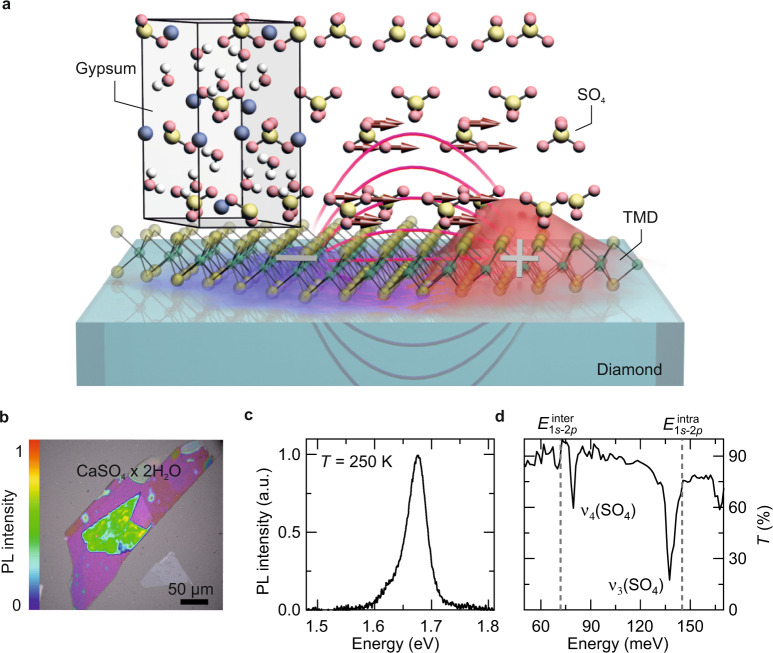
Conceptual idea of strong exciton–phonon proximity coupling. **a** Illustration of interlayer exciton–phonon coupling at the atomic interface of a TMD/gypsum heterostructure. The transient dipole field (magenta curves) of the internal 1*s*–2*p* excitonic transition, represented by a snapshot of the exciton wavefunction during excitation (red and blue surface), can effectively couple to the dipole moment of the infrared active vibrational modes in gypsum (red arrows). **b** Optical micrograph of the monolayer WSe_2_ covered by 100 nm of gypsum. Clear photoluminescence can be observed in WSe_2_ (light green area) after photoexciting the heterostructure at a photon energy of 2.34 eV. **c** Photoluminescence spectrum of a gypsum-covered WSe_2_ ML on a diamond substrate at 250 K, showing a prominent 1*s* A exciton resonance at 1.67 eV. **d** Transmission spectrum of the gypsum layer. The dips at 78 and 138 meV correspond to the vibrational $\nu_4$ and $\nu_3$ modes in gypsum. Dashed vertical line: 1*s*–2*p* resonance of K–K ($E_{1s - 2p}^{{\mathrm{K}} - {\mathrm{K}}}$) excitons in a WSe_2_ monolayer.

Here, we use 2D WSe_2_/gypsum (CaSO_4_·2H_2_O) heterostructures as model systems to demonstrate proximity-induced hybridization between phonons and electrically neutral excitons up to the strong-coupling regime. We tune a Coulomb-mediated quantization energy—the internal 1*s*–2*p* Lyman transition of excitons in WSe_2_—in resonance with polar phonon modes in a gypsum cover layer (Fig. 1a) to create new hybrid excitations called Lyman polarons, which we directly resolve with phase-locked few-cycle mid-infrared (MIR) probe pulses. Engineering the spatial shape of the exciton wavefunction at the atomic scale allows us to manipulate the remarkably strong exciton–phonon coupling and to induce a crosstalk between energetically remote electronic and phononic modes.

## Results

### Rydberg spectroscopy of Lyman polarons

We fabricated three classes of heterostructures—a WSe_2_ ML, a 3R-stacked WSe_2_ bilayer (BL), and a WSe_2_/WS_2_ (tungsten disulfide) heterobilayer (see Supplementary Note 1)—by mechanical exfoliation and all-dry viscoelastic stamping (see “Methods”). All samples were covered with a mechanically exfoliated gypsum layer and transferred onto diamond substrates. Figure 1b shows an exemplary optical micrograph of the WSe_2_ ML/gypsum heterostructure, where strong photoluminescence can be observed from the WSe_2_ ML, attesting to the radiative recombination of 1*s* A excitons (Fig. 1c). The MIR transmission spectrum of gypsum (Fig. 1d) features two absorption peaks caused by the vibrational $\nu_4$ and $\nu_3$ modes of the ${\mathrm{SO}}_4$ tetrahedral groups at 78 and 138 meV, respectively (see “Methods”). These modes are spectrally close to the internal resonance between the orbital 1*s* and 2*p* states of excitons in WSe_2_^21,22^ and are, thus, ideal for exploring the polaron physics that arises from the proximity-induced exciton–phonon coupling at the van der Waals interface. If the coupling strength exceeds the linewidth of both modes one may even expect exciton–phonon hybridization as the excitonic Lyman transition is resonantly dressed by the spatially nearby phonon field (Fig. 1a). In this proximity-induced strong-coupling scenario, Lyman polarons would emerge as new eigenstates of mixed electronic and structural character.

**Fig. 2:**
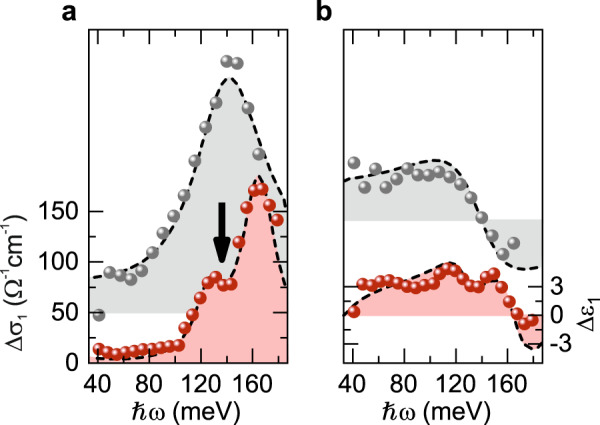
Pump-induced dielectric response of WSe_2_/hBN and WSe_2_/gypsum heterostructures. **a**,**b** Pump-induced changes of the real part of the optical conductivity $\Delta \sigma _1$ (**a**) and the dielectric function $\Delta \varepsilon _1$ (**b**) as a function of the probe photon energy for different heterostructures at *t*_pp_ = 0 ps following resonant femtosecond photogeneration of 1*s* A excitons. Gray spheres: photoinduced dielectric response of a WSe_2_ ML/hBN heterostructure. Red spheres: photoinduced dielectric response of a WSe_2_ ML/gypsum heterostructure. The data are vertically offset for clarity. The dashed lines are fits to the experimental data based on the theoretical model in Eq. (1), by setting *V*_1_ and *V*_2_ to zero. The arrows indicate the characteristic dip in $\Delta \sigma _1$ arising from the strong exciton–phonon coupling.

In the experiment, we interrogate the actual spectrum of low-energy elementary excitations by a phase-locked MIR pulse. The transmitted waveform is electro-optically sampled at a variable delay time, *t*_pp_, after resonant creation of 1*s* A excitons in the K valleys of WSe_2_ by a 100 fs near-infrared pump pulse (see “Methods”). A Fourier transform combined with a Fresnel analysis directly reveals the full dielectric response of the nonequilibrium system (see “Methods”). The pump-induced change of the real part of the optical conductivity, $\Delta \sigma _1$, and of the dielectric function, $\Delta \varepsilon _1$, describe the absorptive and inductive responses, respectively. The dielectric response of a photoexcited WSe_2_ ML covered with hBN at *t*_pp_ = 0 ps (Fig. 2, gray spheres) is dominated by a maximum in $\Delta \sigma _1$ (Fig. 2a) and a corresponding zero crossing in $\Delta \varepsilon _1$ at a photon energy of 143 meV (Fig. 2b). This resonance matches with the established internal 1*s*–2*p* Lyman transition in hBN-covered WSe_2_ MLs^21^ and lies well below the *E*_1u_ phonon mode in hBN (~172 meV, see “Methods”).

In marked contrast, $\Delta \sigma _1$ features a distinct mode splitting for the WSe_2_/gypsum heterostructure (Fig. 2a, red spheres). The two peaks and corresponding dispersive sections in $\Delta \varepsilon _1$ (Fig. 2b, red spheres) are separated by ~35 meV and straddle the internal 1*s*–2*p* Lyman resonance of the WSe_2_/hBN heterostructure. Interestingly, each peak is much narrower than the bare 1*s*–2*p* transition in the WSe_2_/hBN heterostructure. Since the background dielectric constants (neglecting phonons) of gypsum and hBN are similar, the bare 1*s*–2*p* Lyman resonance in a gypsum-covered WSe_2_ ML is expected to appear at an energy close to 143 meV, which gives rise to only a small detuning (Δ*E* ≈ 5 meV) to the vibrational $\nu_3$ mode in gypsum (138 meV). The prominent splitting of $\Delta \sigma _1$ of ~35 meV in the WSe_2_/gypsum heterostructure clearly exceeds the detuning energy and, thus, implies that the two new resonances are indeed Lyman polarons caused by strong-coupling.

### Interlayer exciton–phonon hybridization

Hybridization between the intra-excitonic resonance and a lattice phonon across the van der Waals interface should lead to a measurable anticrossing signature. To test this hypothesis, we perform similar experiments on the WSe_2_ BL/gypsum heterostructure, where the intra-excitonic transition can be tuned through the phonon resonance. Strong interlayer orbital hybridization in the WSe_2_ BL shifts the conduction band minimum from the K points to the Λ points, leading to the formation of K–Λ excitons (${\mathrm{X}}^{{\mathrm{K}} - {{\Lambda }}}$) with wavefunctions delocalized over the top and bottom layer^22,23^. Such interlayer orbital hybridization, which is also commonly observed in other 2D transition metal dichalcogenide (TMD) heterostructures^1–5,8,9,24^, renders the internal 1*s*–2*p* Lyman transition more susceptible to many-body Coulomb renormalization than in a single ML. This offers a unique opportunity to tune the intra-excitonic resonance from 87 to 69 meV by merely increasing the excitation fluence from 5 to 36 µJ cm^−2^ (see Supplementary Note 2).

**Fig. 3:**
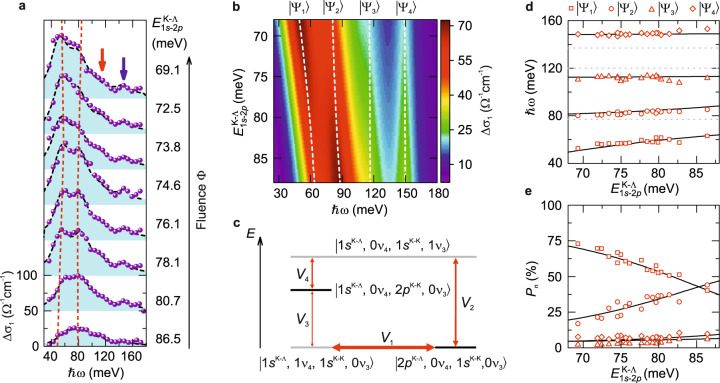
Anticrossing in interlayer exciton–phonon quantum hybridization. **a** Experimentally observed pump-induced changes of $\Delta \sigma _1$ (*t*_pp_ = 3 ps, *T* = 260 K) of a 3R-stacked WSe_2_ bilayer covered with few-layer gypsum, for different excitation fluences Φ indicated on the right. When Φ is increased from 5 to 36 µJ cm^−2^ (from bottom to top), many-body renormalization shifts the intra-excitonic resonance $E_{1s - 2p}^{{\mathrm{K}} - {{\Lambda }}}$ from above to below the *ν*_4_ phonon resonance, unveiling exciton–phonon anticrossing. The red dashed lines are guides to the eyes for the peak position of $\left| {{{\Psi }}_1} \right\rangle$ and $\left| {{{\Psi }}_2} \right\rangle$. The excellent agreement between theoretical simulation (black dashed lines) and experimental data (solid spheres) across all $E_{1s - 2p}^{{\mathrm{K}} - {{\Lambda }}}$ confirms the interlayer exciton–phonon hybridization. **b** Two-dimensional plot of the simulated $\Delta \sigma _1$ spectra based on the Hamiltonian shown in Eq. (1) as a function of $E_{1s - 2p}^{{\mathrm{K}} - {{\Lambda }}}$. The color scale, vertical axis, and horizontal axis represent $\Delta \sigma _1$, $E_{1s - 2p}^{{\mathrm{K}} - {{\Lambda }}}$, and the probe photon energy, respectively. Dashed white lines indicate the energies of Lyman polaron eigenstates ($\left| {{{\Psi }}_n} \right\rangle$, *n* = 1, 2, 3, 4). **c** Illustration of the effective coupling between different excitonic states (black lines) and phonon states (gray lines). When the zero-phonon 1*s*–2*p* transition energy of the K–Λ exciton state $\left| {2p^{{\mathrm{K}} - {{\Lambda }}},\,0\nu_4,1s^{{\mathrm{K}} - {\mathrm{K}}},0\nu_3} \right\rangle$ is tuned across the $\nu_4$ state $\left| {1s^{{\mathrm{K}} - {{\Lambda }}},\,1\nu_4,1s^{{\mathrm{K}} - {\mathrm{K}}},0\nu_3} \right\rangle$ (denoted by the thick red arrow) anticrossing occurs. **d**, **e** Solid lines show the calculated energies of the Lyman polaron eigenstates (**d**) and projection (*P*_*n*_) of the eigenstates to the bare $\left| {2p^{{\mathrm{K}} - {{\Lambda }}},\,0\nu_4,1s^{{\mathrm{K}} - {\mathrm{K}}},0\nu_3} \right\rangle$ state (**e**) as a function of $E_{1s - 2p}^{{\mathrm{K}} - {{\Lambda }}}$. The dashed lines in **d** show the energies of $\left| {1s^{{\mathrm{K}} - {{\Lambda }}},\,0\nu_4,2p^{{\mathrm{K}} - {\mathrm{K}}},0\nu_3} \right\rangle$ (115 meV), $\left| {1s^{{\mathrm{K}} - {{\Lambda }}},\,1\nu_4,1s^{{\mathrm{K}} - {\mathrm{K}}},0\nu_3} \right\rangle$ (78 meV) and $\left| {1s^{{\mathrm{K}} - {{\Lambda }}},\,0\nu_4,1s^{{\mathrm{K}} - {\mathrm{K}}},1\nu_3} \right\rangle$ (138 meV). The symbols in **d**, **e** correspond to $\left| {{{\Psi }}_n} \right\rangle$ and were obtained from fits to the measured $\Delta \sigma _1$ spectra.

Figure 3a displays the MIR response of the WSe_2_ BL/gypsum heterostructure at *t*_pp_ = 3 ps and various excitation densities. Strikingly, we observe a distinct anticrossing near the 1*s*–2*p* Lyman transition of K–Λ excitons in the WSe_2_ BL and the $\nu_4$ mode of gypsum upon increasing the excitation density. This is unequivocal evidence of hybridization of exciton and phonon modes across the atomic interface. In addition, the absorption for all excitation densities exhibits a discernible shoulder at a photon energy of ~115 meV (Fig. 3a, red arrow), which is very close to the 1*s*–2*p* resonance of K–K excitons (${\mathrm{X}}^{{\mathrm{K}} - {\mathrm{K}}}$)^22^. Such a transition is indeed expected to occur at short delay times *t*_pp_ < 1 ps, when the bound electron–hole pairs are prepared in the K valleys through direct interband excitation. However, the subtle interplay between 2D confinement and interlayer orbital overlap in the BL gives rise to a complex energy landscape^1–5,8,9,24^, where the lowest-energy exciton state is given by K–Λ species. Thus, sub-picosecond thermalization of the electron to Λ valleys via intervalley scattering^22,23,25^ should render the 1*s*–2*p* transition of K–K excitons weak. Yet, we clearly observe its spectral signature during the entire lifetime (see Supplementary Note 3). In addition, a new absorption band appears above the $\nu_3$ resonance of gypsum at an energy of ~150 meV (Fig. 3a, blue arrow). Its spectral position is nearly independent of the excitation density. We will show next that these surprising observations hallmark interlayer exciton–phonon hybridization involving as many as two phonon and two exciton resonances across the atomic interface, at once.

The dominant anticrossing feature in Fig. 3a occurs at an energy close to the $\nu_4$ mode of gypsum (78 meV) and the 1*s*–2*p* resonance of K–Λ excitons in WSe_2_ (69–87 meV, depending on the excitation fluence), while additional optical transitions emerge at energies close to the $\nu_3$ mode (138 meV) and the 1*s*–2*p* resonance of K–K excitons (115 meV). Therefore, we consider how the 1*s*–2*p* transition of K-Λ and K–K excitons (see Supplementary Note 4) hybridize with $\nu_3$ and $\nu_4$ phonons in gypsum. The electron–phonon interaction is commonly described by the Fröhlich Hamiltonian, which is linear in the phonon creation and annihilation operators and couples only states differing by one optical phonon^26^. The energetically lowest excited states of the uncoupled system, in which only one of the two exciton species or one of the two phonons is excited, can be denoted as $\left| {2p^{{\mathrm{K}} - \Lambda },\,0\nu_4,\,1s^{{\mathrm{K}} - {\mathrm{K}}},\,0\nu_3} \right\rangle$, $\left| {1s^{{\mathrm{K}} - {{\Lambda }}},\,1\nu_4,\,1s^{{\mathrm{K}} - {\mathrm{K}}},0\nu_3} \right\rangle$, $\left| {1s^{{\mathrm{K}} - {{\Lambda }}},\,0\nu_4,\,2p^{{\mathrm{K}} - {\mathrm{K}}},\,0\nu_3} \right\rangle$, $\left| {1s^{{\mathrm{K}} - {{\Lambda }}},\,0\nu_4,\,1s^{{\mathrm{K}} - {\mathrm{K}}},\,1\nu_3} \right\rangle$. The coupling between different states is illustrated in Fig. 3c. Using these basis vectors, we derive an effective Hamiltonian

1 $$H_{{\mathrm{eff}}} = \left( \begin{array}{*{20}{c}} {E_{1s - 2p}^{{\mathrm{K}} - {{\Lambda }}}} & {V_1} & 0 & {V_2} \\ {V_1} & {E_{{\mathrm{ph}}}^{\nu_4}} & {V_3} & 0 \\ 0 & {V_3} & {E_{1s - 2p}^{{\mathrm{K}} - {\mathrm{K}}}} & {V_4} \\ {V_2} & 0 & {V_4} & {E_{{\mathrm{ph}}}^{\nu_3}} \end{array} \right).$$

Here, $E_{1s - 2p}^{{\mathrm{K}} - {{\Lambda }}}$ $( {E_{1s - 2p}^{{\mathrm{K}} - {\mathrm{K}}}})$ and $E_{{\mathrm{ph}}}^{\nu_4}$ $( {E_{{\mathrm{ph}}}^{\nu_3}} )$ denote the 1*s*–2*p* resonance energy of K–Λ (K–K) excitons and the energy of the $\nu_4$ ($\nu_3$) mode, respectively, whereas $V_1$, $V_2$, $V_3$, and $V_4$ describe the exciton–phonon coupling constants (Fig. 3c, red arrows). At exciton densities for which $E_{1s - 2p}^{{\mathrm{K}} - {{\Lambda }}}$ is tuned through $E_{{\mathrm{ph}}}^{\nu_4}$, the Hamiltonian shows that the resonant exciton–phonon hybridization leads to an avoided crossing. Quantitative comparison between experiment and theory can be achieved by directly solving the effective Hamiltonian and yields four new hybrid states ($\left| {{{\Psi }}_n} \right\rangle$, *n* = 1, 2, 3, 4) that consist of a superposition of the basis modes.

Figure 3b displays a 2D map of the simulated optical conductivity of the new polaron eigenstates $\left| {{{\Psi }}_n} \right\rangle$ as a function of the probe energy ($\hbar \omega$) and the position of $E_{1s - 2p}^{{\mathrm{K}} - {{\Lambda }}}$. Since the excitons in WSe_2_ are largely thermalized as K–Λ species on a sub-picosecond scale, the oscillator strength of the resulting polarons observed thereafter depends on their projection $P_n = \left\langle {{{\Psi }}_n|2p^{{\mathrm{K}} - {{\Lambda }}},0\nu_4,1s^{{\mathrm{K}} - {\mathrm{K}}},\,0\nu_3} \right\rangle$ onto the bare zero-phonon K–Λ exciton (see Supplementary Note 3). To validate our model, we fit the simulated optical conductivity to the experimental data (Fig. 3a). Again, we set $E_{{\mathrm{ph}}}^{\nu_4}$ = 78 meV and $E_{{\mathrm{ph}}}^{\nu_3}$ = 138 meV (see Fig. 1d), and $E_{1s - 2p}^{{\mathrm{K}} - {\mathrm{K}}}$ = 115 ± 5 meV (ref. ^22^), while $E_{1s - 2p}^{{\mathrm{K}} - {{\Lambda }}}$ and the oscillator strength of the 1*s*–2*p* transition of the K–Λ exciton serve as fit parameters. For oscillator strengths similar to published values in ref. ^22^, the numerical adaption yields excellent agreement between theory and experiment and reproduces all optical transitions (Fig. 3a) and the prominent anticrossing (Fig. 3d).

The coupling constants retrieved from fitting the model to the experimental data amount to $V_1 \approx V_3 =$ 20 ± 2 meV and $V_2 \approx V_4 =$ 31 ± 2 meV (see Supplementary Note 3), even exceeding values reported in quantum dots^27^. This result is remarkable given that in our experiments strong-coupling is only achieved by proximity across the van der Waals interface. The ratio $\frac{{V_2}}{{V_1}} \sim \frac{{V_4}}{{V_3}} \sim \sqrt 2$ qualitatively reflects the relative dipole moments of the $\nu_4$ and $\nu_3$ modes (see Supplementary Note 3). Our analysis also allows us to assign the high-frequency features in Fig. 3a to $\left| {{{\Psi }}_3} \right\rangle$ and $\left| {{{\Psi }}_4} \right\rangle$. Even when the K–K excitons are weakly populated at *t*_pp_ = 3 ps and the $\nu_3$ phonon resonance is far-detuned from the 1*s*–2*p* resonance of K–Λ excitons, the strong-coupling scenario allows for these Lyman polarons to emerge. In addition, by increasing the excitation density, many-body Coulomb correlations shift the bare 1*s*–2*p* resonance of K–Λ excitons and thereby modify the Lyman composition of $\left| {{{\Psi }}_n} \right\rangle$, as shown in Fig. 3e. For example, for $E_{1s - 2p}^{{\mathrm{K}} - {{\Lambda }}}$ = 86.5 meV, $\left| {{{\Psi }}_1} \right\rangle$ consists of 40% (6%) 1*s*–2*p* Lyman transition of the K–Λ (K–K) exciton and 10% (44%) $\nu_3$ ($\nu_4$) phonon (see Supplementary Note 3).

### Shaping the interlayer exciton–phonon coupling strength

**Fig. 4:**
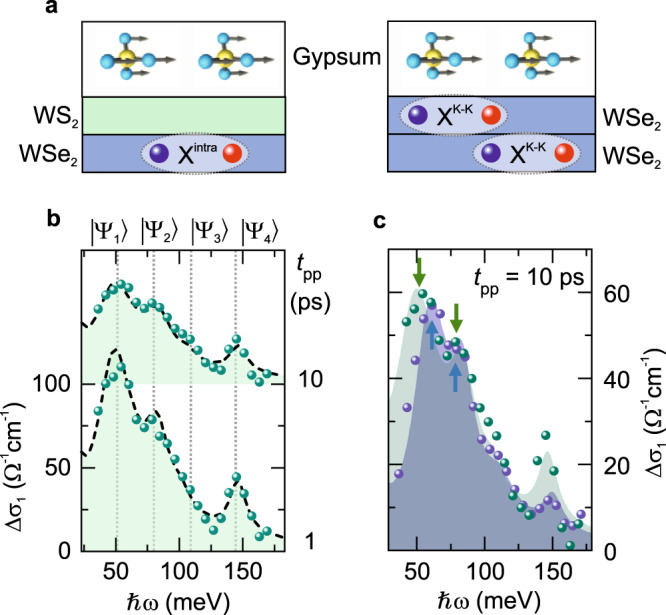
MIR response of the WSe_2_/WS_2_/gypsum heterostructure and comparison to the WSe_2_ BL/gypsum heterostructure. **a** Schematic of the spatial distribution of the excitons in the WSe_2_/WS_2_/gypsum (left) and the WSe_2_ BL/gypsum (right) heterostructure. The relative distance of intralayer excitons (${\mathrm{X}}^{{\mathrm{intra}}}$ in the WSe_2_/WS_2_ heterostructure and ${\mathrm{X}}^{{\mathrm{K}} - {\mathrm{K}}}$ in the WSe_2_ BL) to the vibrational modes in gypsum yields different coupling strength. **b** Pump-induced change of the real part of the optical conductivity Δ*σ*_1_ for different *t*_pp_ for a WSe_2_/WS_2_/gypsum heterostructure (*T* = 230 K). Green spheres: experimental data. Black dashed line: theoretical simulation. Gray dotted lines indicate the spectral positions of $\left| {{{\Psi }}_n} \right\rangle$. **c** Comparison between Δ*σ*_1_ of the WSe_2_/WS_2_/gypsum (green) and the WSe_2_ BL/gypsum (blue) heterostructure at *t*_pp_ = 10 ps. Spheres: experimental data. Shaded areas: theoretical simulation. Green and blue arrows indicate the positions of $\left| {{{\Psi }}_1} \right\rangle$ and $\left| {{{\Psi }}_2} \right\rangle$.

The interlayer exciton–phonon hybridization can be custom-tailored by engineering the spatial overlap of exciton and phonon wavefunctions on the atomic scale. To demonstrate this possibility, we create spatially well-defined intra- (${\mathrm{X}}^{{\mathrm{intra}}}$) and interlayer exciton (${\mathrm{X}}^{{\mathrm{inter}}}$) phases by interfacing the WSe_2_ ML with a WS_2_ ML in a WSe_2_/WS_2_/gypsum heterostructure. Unlike in the WSe_2_ BL/gypsum heterostructure, the intralayer excitons in WSe_2_ are now spatially separated from gypsum by the WS_2_ ML (Fig. 4a). Ultrafast charge separation at the interface between WSe_2_ and WS_2_ depletes the Lyman resonance of ${\mathrm{X}}^{{\mathrm{intra}}}$, while the transition of ${\mathrm{X}}^{{\mathrm{inter}}}$ emerges on the sub-picosecond timescale^21^. Figure 4b shows $\Delta \sigma _1$ of the WSe_2_/WS_2_/gypsum heterostructure at *t*_pp_ = 1 and 10 ps. The MIR response arising from exciton–phonon coupling is qualitatively similar to that observed in the WSe_2_ BL/gypsum heterostructure. This is partly because both the inter- ($E_{1s - 2p}^{{\mathrm{inter}}}$ = 69 meV) and intralayer ($E_{1s - 2p}^{{\mathrm{intra}}}$ = 114 meV) 1*s*–2*p* resonance in the WSe_2_/WS_2_ heterostructure are similar to $E_{1s - 2p}^{{\mathrm{K}} - {{\Lambda }}}$ (69–87 meV, depending on the excitation fluence) and $E_{1s - 2p}^{{\mathrm{K}} - {\mathrm{K}}}$ (115 meV) in the WSe_2_ BL, respectively. However, a direct comparison between $\Delta \sigma _1$ of both systems reveals a strong enhancement of the oscillator strength of the $\left| {{{\Psi }}_4} \right\rangle$ mode and larger splitting between $\left| {{{\Psi }}_1} \right\rangle$and $\left| {{{\Psi }}_2} \right\rangle$ in the WSe_2_/WS_2_/gypsum heterostructure (Fig. 4c). By fitting the experimental data with our coupling model, we found that the large oscillator strength of $\left| {{{\Psi }}_4} \right\rangle$ arises from enhanced phonon coupling to ${\mathrm{X}}^{{\mathrm{inter}}}$ of *V*_1_ = 22 ± 2 meV and *V*_2_ = 36 ± 2 meV, which may be related with the dipolar nature of interlayer excitons. Meanwhile, the interlayer exciton–phonon coupling strength, which results directly from ${\mathrm{X}}^{{\mathrm{intra}}}$ confined in the WSe_2_ layer, amounts to *V*_3_ = 16 ± 2 meV and *V*_4_ = 25 ± 2 meV (see Supplementary Note 5). The reduction of coupling strength, *V*_3_ and *V*_4_, by at least 20% compared to the WSe_2_ BL/gypsum case can be attributed to the atomically small spatial separation of the WSe_2_ ML from gypsum, which illustrates how exciton–phonon interaction could be fine-tuned in search for new phases of matter.

## Discussion

Our results reveal that even charge-neutral quasiparticles can interact with phonons across a van der Waals interface in the strong-coupling limit. Controlling excitonic wavefunctions at the atomic length scale can modify the coupling strength. We expect important implications for the study of polaron physics with charged and neutral excitations in a wide range of atomically thin strongly correlated electronic systems. In particular, polarons are known to play a crucial role in the formation of charge-density waves in Mott insulators and Cooper pairs in superconductors^16,19^. Moreover, excitons in TMD heterostructures embody important properties arising from the valley degree of freedom and can be engineered from topologically protected edge states of moiré superlattices^1–4,13,14,28–30^. In the future, it might, thus, even become possible to transfer fascinating aspects of chirality and nontrivial topology to polaron transport.

## Methods

### Sample preparation

All heterostructure compounds were exfoliated mechanically from a bulk single crystal using the viscoelastic transfer method^31^. We used gypsum and hBN as dielectric cover layers. The vibrational $\nu_4$ and $\nu_3$ modes of the ${\mathrm{SO}}_4$ tetrahedral groups^32^ in gypsum (Fig. 1d) are close to the internal 1*s*–2*p* transition of excitons in WSe_2_. In contrast, the prominent *E*_1u_ mode in hBN at 172 meV^33^ is far-detuned from the internal 1*s*–2*p* exciton transition in the WSe_2_ layer. The exfoliated gypsum, hBN, and TMD layers were inspected under an optical microscope and subsequently stacked on top of each other on a diamond substrate with a micro-positioning stage. To remove any adsorbates, the samples were annealed at a temperature of 150 °C and a pressure of 1 × 10^−5^ mbar for 5 h. The twist angle of the WSe_2_ BL was ensured by the tear and stack method: Starting from an extremely large exfoliated monolayer, only half of it is transferred onto the substrate. Consequently, transferring the remaining part of the ML onto the diamond substrate yields a perfectly aligned WSe_2_ BL.

### Ultrafast pump-probe spectroscopy

Supplementary Figure 6a depicts a schematic of the experimental setup. A home-built Ti:sapphire laser amplifier with a repetition rate of 400 kHz delivers ultrashort 12-fs NIR pulses. The output of the beam is divided into three branches. A first part of the laser output is filtered by a bandpass filter with a center wavelength closed to the interband 1*s* A exciton transition in the WSe_2_ layer, and a bandwidth of 9 nm, resulting in 100-fs pulses. Another part of the laser pulse generates single-cycle MIR probe pulses via optical rectification in a GaSe or an LGS crystal (NOX1). The probe pulse propagates through the sample after a variable delay time *t*_pp_. The electric field waveform of the MIR transient and any changes induced by the nonequilibrium polarization of the sample are fully resolved by electro-optic sampling utilizing a second nonlinear crystal (NOX2) and subsequent analysis of the field-induced polarization rotation of the gate pulse. Supplementary Figure 6b shows a typical MIR probe transient as a function of the electro-optic sampling time *t*_eos_. The MIR probe pulse is centered at a frequency of 32 THz with a full-width at half-maximum of 18 THz (Supplementary Fig. 6c, black curve) and a spectral phase that is nearly flat (Supplementary Fig. 6c, blue curve). Using serial lock-in detection, we simultaneously record the pump-induced change Δ*E*(*t*_eos_) and a reference *E*_ref_(*t*_eos_) of the MIR electric field as function of *t*_eos_.

### Extracting the dielectric response function

To extract the pump-induced change of the dielectric function of our samples with ultrafast NIR pump-MIR probe spectroscopy, we use serial lock-in detection. Hereby, a first lock-in amplifier records the electro-optic signal of our MIR probe field. Due to the modulation of the optical pump, the transmitted MIR probe field varies by the pump-induced change ${{\Delta }}E( {t_{{\mathrm{eos}}},t_{{\mathrm{pp}}}} )$. This quantity is read out in a second lock-in amplifier at the modulation frequency of the pump. Simultaneously, the electro-optic signal is averaged in an analog low-pass to obtain a reference signal $E_{{\mathrm{ref}}}( {t_{{\mathrm{eos}}}} ) = \frac{1}{2}( E_{{\mathrm{ex}}}( {t_{{\mathrm{eos}}},t_{{\mathrm{pp}}}} ) + E_{{\mathrm{eq}}}( {t_{{\mathrm{eos}}}} ))$, where $E_{{\mathrm{eq}}}\left( {t_{{\mathrm{eos}}}} \right)$ is the signal after transmission through the sample in thermal equilibrium and $E_{{\mathrm{ex}}}( {t_{{\mathrm{eos}}},t_{{\mathrm{pp}}}} ) = E_{{\mathrm{eq}}}( {t_{{\mathrm{eos}}}} ) + {{\Delta }}E( {t_{{\mathrm{eos}}},t_{{\mathrm{pp}}}} )$ is the signal after transmission through the excited sample at $t_{{\mathrm{pp}}}$. From these quantities $E_{{\mathrm{eq}}}\left( {t_{{\mathrm{eos}}}} \right)$ and $E_{{\mathrm{ex}}}\left( {t_{{\mathrm{eos}}}} \right)$ are directly extracted. Subsequently, a Fourier transform for a fixed $t_{{\mathrm{pp}}}$ yields $E_{{\mathrm{eq}}}\left( \omega \right)$ and $E_{{\mathrm{ex}}}( {\omega ,t_{{\mathrm{pp}}}} )$, which in turn provides us with the complex-valued field transfer coefficient of our layered structure

2 $$T_{{\mathrm{pr}}}\left( {\omega ,t_{{\mathrm{pp}}}} \right) = T_{{\mathrm{pi}}}\left( {\omega ,t_{{\mathrm{pp}}}} \right) T_{{\mathrm{eq}}}\left( \omega \right) = \frac{{E_{{\mathrm{ex}}}\left( {\omega ,t_{{\mathrm{pp}}}} \right)}}{{E_{{\mathrm{eq}}}\left( \omega \right)}} T_{{\mathrm{eq}}}\left( \omega \right),$$

where $T_{{\mathrm{eq}}}\left( \omega \right)$ is the equilibrium field transmission coefficient and $T_{{\mathrm{pi}}}( {\omega ,t_{{\mathrm{pp}}}} )$ denotes the pump-induced change thereof. These quantities are completely defined by the equilibrium dielectric function $\varepsilon \left( \omega \right)$ and its pump-induced change ${{\Delta }}\varepsilon \left( {\omega ,t_{{\mathrm{pp}}}} \right)$. By using the established optical transfer-matrix formalism^34^, we express the experimentally measured $T_{{\mathrm{pr}}}( {\omega ,t_{{\mathrm{pp}}}} )$ with the dielectric function. Finally, we insert the known equilibrium dielectric function and numerically invert the optical transfer-matrix formalism to extract the coveted quantity ${{\Delta }}\varepsilon ( {\omega ,t_{{\mathrm{pp}}}} )$ discussed in the main text. Owing to the extremely thin sample thickness, challenges associated with Fabry–Perot resonances are unproblematic here and the inversion algorithm is especially stable and quantitatively reliable.

## Supplementary information

Supplementary Information

## Data Availability

The datasets generated during and/or analyzed during the current study are available from the corresponding author on reasonable request to give guidance to the interested party.
