# Supplementary material for: Proximity control of interlayer exciton-phonon hybridization in van der Waals heterostructures
Source: Nat Commun. 2021 Mar 19;12:1719. doi: 10.1038/s41467-021-21780-6 (PMC7979927; doi:10.1038/s41467-021-21780-6)
Supplement: Supplementary file 1 — Supplementary Information [file 41467_2021_21780_MOESM1_ESM.pdf]

Supplementary Information for

Proximity control of interlayer exciton-phonon hybridization in van der Waals heterostructures

Merkl *et al.*

Supplementary Note 1

The samples are fabricated as stated in the methods section. Supplementary Fig. 1 shows an optical micrograph of the WSe_2/WS_2/gypsum (**a**) and the WSe_2 BL/gypsum heterostructure (**b**). The WSe_2 BL and WSe_2/WS_2 heterostructure are highlighted by the black frame.

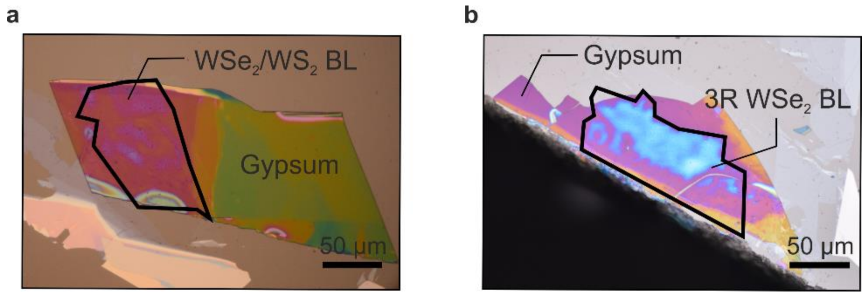

Supplementary Figure 1 | Optical micrograph of the TMD/gypsum samples. Optical microscope images of the WSe_2/WS_2/gypsum (**a**) and the BL/gypsum heterostructure (**b**) on diamond substrates. The black frames indicate the overlap region of the TMD monolayers. Each of the bilayers is covered by a thin capping layer of gypsum.

Supplementary Note 2

Supplementary Fig. 2 shows a typical MIR response of an uncovered WSe_2 BL at a pump probe delay time of t_{pp} = 5 ps and an excitation fluence of Φ = 7 μJcm^{-2}. For all fluences, a clear maximum in the real part of the optical conductivity $\Delta\sigma_1$ and a corresponding dispersive feature in the real part of the dielectric function $\Delta\varepsilon_1$, is observable, indicative of the dominant internal 1*s*-2*p* Lyman transition of K-Λ excitons. To extract the resonance energy, we fit a phenomenological two-component model to the experimental data[1]. Supplementary Fig. 2c summarizes the transition energies $E_{1s-2p}^{\mathrm{K}-\Lambda}$ in the bare WSe_2 BL at various excitation fluences (red diamonds). Many-body interactions of electron-hole pairs reduce $E_{1s-2p}^{\mathrm{K}-\Lambda}$ in the uncovered WSe_2 from 80 to 64 meV as Φ is increased from 7 to 27 μJcm^{-2}.

The resonance energy $E_{1s-2p}^{\mathrm{K}-\Lambda}$ in the WSe_2 BL/gypsum heterostructure was extracted analogously by fitting the experimental data with the effective coupling model (see eq. 1 in the main text). Supplementary Fig. 2c depicts $E_{1s-2p}^{\mathrm{K}-\Lambda}$ of the WSe_2 BL/gypsum heterostructure (purple spheres) at various excitation fluences, and follows the trend of the bare WSe_2 BL.

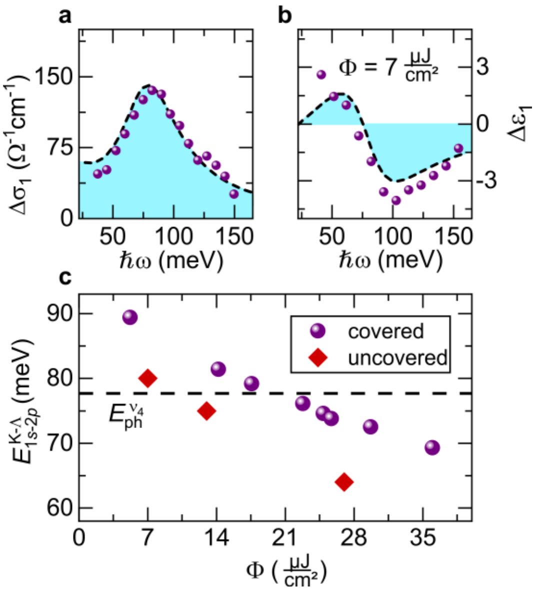

Supplementary Figure 2 | Many-body renormalization effects on the bare K-Λ exciton state. a,**b**, Pump-induced changes of the real part of the optical conductivity $\Delta\sigma_1$ (**a**) and real part of the dielectric function $\Delta\varepsilon_1$ (**b**) as a function of the photon energy. The data were recorded on an uncovered 3R stacked WSe_2 bilayer at a pump-probe delay time of t_{pp} = 5 ps and a temperature of 5 K. The purple spheres indicate the experimental data and the blue shaded areas represent the fit with a two-component model. **c**, Bare resonance energies $E_{1s-2p}^{\mathrm{K}-\Lambda}$ of the internal 1*s*-2*p* Lyman transition of an uncovered WSe_2 BL (red diamonds) and the WSe_2 BL/gypsum heterostructure (purple spheres) as a function of the excitation fluence Φ.

Supplementary Note 3

Strong interlayer exciton-phonon coupling can be described by the effective Hamiltonian H_{eff} given in eq. (1) in the main text. The resonance energies of the bare SO_4 phonon modes determined from the transmission measurement (Fig. 1d) are $E_{\mathrm{ph}}^{v_4}$ = 78 meV and $E_{\mathrm{ph}}^{v_3}$ = 138 meV, are in good agreement with literature[2]. The diagonalization of H_{eff} yields the eigenenergies E_n and the eigenmodes $|\Psi_n\rangle$ of the excitonic Lyman-polaron states. To extract the exciton-phonon coupling constants V_1, V_2, V_3, and V_4 we numerically adapt our effective coupling model to the experimental data. Therefore, we simulate the pump-induced change of the dielectric function $\Delta\varepsilon(\omega) = \Delta\varepsilon_1(\omega) + i\Delta\sigma_1(\omega)/(\varepsilon_0\omega)$ according to:

$$\Delta\varepsilon(\omega) = \sum_{n=1}^{4}\left[\frac{A_n}{\left(\frac{E_n^2}{\hbar^2}-\omega^2-i\omega\gamma_n\right)}\right] - \frac{A_{\mathrm{fc}}}{(\omega^2-i\omega\gamma_{\mathrm{fc}})} \qquad \text{(S1)}$$

where four Lorentzian functions with oscillator strength A_n and linewidth γ_n represent the excitonic Lyman-polaron absorption bands and the last term accounts for a Drude-like response of a weak background of unbound electron hole pairs with oscillator strength A_{fc} and linewidth γ_{fc} typically present in TMD heterostructures[3,4]. A_n and γ_n can be determined by the projection matrix of the new eigenstates to the bare states

$$P = \begin{pmatrix} \langle\Psi_1|2p^{\mathrm{K-\Lambda}},0v_4,1s^{\mathrm{K-K}},0v_3\rangle & \langle\Psi_2|2p^{\mathrm{K-\Lambda}},0v_4,1s^{\mathrm{K-K}},0v_3\rangle & \langle\Psi_3|2p^{\mathrm{K-\Lambda}},0v_4,1s^{\mathrm{K-K}},0v_3\rangle & \langle\Psi_4|2p^{\mathrm{K-\Lambda}},0v_4,1s^{\mathrm{K-K}},0v_3\rangle \\ \langle\Psi_1|1s^{\mathrm{K-\Lambda}},1v_4,1s^{\mathrm{K-K}},0v_3\rangle & \langle\Psi_2|1s^{\mathrm{K-\Lambda}},1v_4,1s^{\mathrm{K-K}},0v_3\rangle & \langle\Psi_3|1s^{\mathrm{K-\Lambda}},1v_4,1s^{\mathrm{K-K}},0v_3\rangle & \langle\Psi_4|1s^{\mathrm{K-\Lambda}},1v_4,1s^{\mathrm{K-K}},0v_3\rangle \\ \langle\Psi_1|1s^{\mathrm{K-\Lambda}},0v_4,2p^{\mathrm{K-K}},0v_3\rangle & \langle\Psi_2|1s^{\mathrm{K-\Lambda}},0v_4,2p^{\mathrm{K-K}},0v_3\rangle & \langle\Psi_3|1s^{\mathrm{K-\Lambda}},0v_4,2p^{\mathrm{K-K}},0v_3\rangle & \langle\Psi_4|1s^{\mathrm{K-\Lambda}},0v_4,2p^{\mathrm{K-K}},0v_3\rangle \\ \langle\Psi_1|1s^{\mathrm{K-\Lambda}},0v_4,1s^{\mathrm{K-K}},1v_3\rangle & \langle\Psi_2|1s^{\mathrm{K-\Lambda}},0v_4,1s^{\mathrm{K-K}},1v_3\rangle & \langle\Psi_3|1s^{\mathrm{K-\Lambda}},0v_4,1s^{\mathrm{K-K}},1v_3\rangle & \langle\Psi_4|1s^{\mathrm{K-\Lambda}},0v_4,1s^{\mathrm{K-K}},1v_3\rangle \end{pmatrix} \qquad \text{(S2)}$$

via $A_n = \sqrt{\sum_{n,m} P_{n,m}\, {A_m^0}^2}$ and $\gamma_n = \sqrt{\sum_{n,m} P_{n,m}\, {\gamma_m^0}^2}$ with the oscillator strength A^0 and linewidth γ^0 of the bare exciton and phonon states. In the WSe_2 BL, the initially photoexcited K-K excitons are thermalized to the K-Λ state on a sub-picosecond time scale. At t_{pp} = 3 ps, the bare oscillator strength reads $A^0 = \left(A_{1s-2p}^{\mathrm{K-\Lambda}}, 0, 0, 0\right)$. Likewise, the bare linewidth reads $\gamma^0 = \left(\gamma_{1s-2p}^{\mathrm{K-\Lambda}}, \gamma_{v_4}, \gamma_{1s-2p}^{\mathrm{K-K}}, \gamma_{v_3}\right)$, where the linewidth of the phonon modes is set to $\gamma_{v_3} = \gamma_{v_4} =$ 10 meV in good agreement with the transmission measurement (Fig. 1d). The $1s$-$2p$ transition linewidths are kept in a typical range of $\gamma_{1s-2p}^{\mathrm{K-\Lambda}}$, $\gamma_{1s-2p}^{\mathrm{K-K}}$ ∈ [35, 55] meV. In addition the bare exciton resonance energies can be restricted to $E_{1s-2p}^{\mathrm{K-\Lambda}}$ ∈ [65, 85] meV and $E_{1s-2p}^{\mathrm{K-K}}$ ∈ [110, 120] meV[3,4]. Accordingly, the number of free parameters is effectively reduced and we

parametrize H_{eff} by means of $A_{1s-2p}^{\text{K}-\Lambda}$ and the coupling constants V_n (n = 1, 2, 3, 4). The adaption of the dielectric function in eq. (S1) to our experimental data is ensured by numerical optimization of the input parameters via the least-squares method. Without any further restrictions, the algorithm converges and shows an excellent agreement between the model and the experimental data, and yields all features observed in the photo-induced change of the dielectric response. Strikingly the optimization procedure independently reproduces the red shift of $E_{1s-2p}^{\text{K}-\Lambda}$ for increasing excitation fluence (Supplementary Fig. 2c). In addition, the ratio $\frac{V_2}{V_1} \sim \frac{V_4}{V_3} \sim \sqrt{2}$ qualitatively reflects the relative oscillator strength of the bare phonon modes $A_{v_4} \sim 2A_{v_3}$ ($A_{v_i} \sim \boldsymbol{p}_{v_i}^2$, i = 3, 4, where $\boldsymbol{p}_{v_i}$ is the dipole moment of v_i phonon modes) in the transmission measurement (Fig. 1d), suggesting a dipole-dipole like interaction ($V_n \propto \boldsymbol{p}_{v_i} \cdot \boldsymbol{p}_{\text{X}}$, where $\boldsymbol{p}_{\text{X}}$ is the dipole moment of internal 1s-2p intraexcitonic transition). This reaffirms the comprehensive agreement of the optimized parameter sets and the experimental observations.

The strength of the hybridization between the excitonic transition and the SO_4 phonon modes depends on the symmetry of the latter. Owing to the 2D nature of the exciton wavefunction, the 1s-2p transition dipole lies within the TMD plane. Therefore, coupling to the out-of-plane phonon modes of SO_4 can be negligibly small, depending on the projection of their dipole moment onto the TMD plane. In contrast, in-plane phonon modes of SO_4, such as the v_3 and v_4 phonon modes discussed in the main text, can couple rather efficiently. Since the energy separation between the in-plane phonon modes of different symmetry is fairly small (~5 meV, ref.[2]) and the 1s-2p transition dipole moment is equally large in all in-plane directions, the exciton-phonon coupling is rather insensitive to the in-plane alignment of the gypsum crystal with respect to the WSe_2 bilayer. Potentially remaining small differences of in-plane modes of order 5 meV would be buried in the spectral resolution of our experiments.

The WSe_2-diamond substrate interaction, in turn, is expected to be negligibly weak. First, the 5.5 eV bandgap of diamond should prevent electronic interaction with the WSe_2 layer. Second, diamond does not feature infrared active phonon resonances in the MIR spectral range[5,6]. These combined effects rule out the exciton-phonon interaction between the WSe_2 and the diamond substrate.

Supplementary Fig. 3 compares our microscopic model and the experimental data of the WSe_2 BL/gypsum heterostructure at t_{pp} = 10 ps and Φ = 23 μJcm^{-2}, yielding an almost perfect agreement. In-particular, our model captures the absorption features near 115 meV, which we assigned to the $|\Psi_3\rangle$ band. This hybrid state is primarily composed of $\left|1s^{\text{K}-\Lambda}, 0v_4, 2p^{\text{K}-\text{K}}, 0v_3\right\rangle$

(Supplementary Fig. 3b), in accordance with the projection of $|\Psi_3\rangle$ to $|1s^{\mathrm{K-\Lambda}}, 0v_4, 2p^{\mathrm{K-K}}, 0v_3\rangle$, and cannot be reproduced if neglecting the coupling of $|1s^{\mathrm{K-\Lambda}}, 0v_4, 2p^{\mathrm{K-K}}, 0v_3\rangle$ with the manifolds of $|1s^{\mathrm{K-\Lambda}}, 1v_4, 1s^{\mathrm{K-K}}, 0v_3\rangle$ and $|1s^{\mathrm{K-\Lambda}}, 0v_4, 1s^{\mathrm{K-K}}, 1v_3\rangle$ in the Hamiltonian (by setting V_3 and V_4 to zero, see dashed line in Supplementary Fig. 3a). The projection P_n of the Lyman polaron states $|\Psi_n\rangle$ onto $|2p^{\mathrm{K-\Lambda}}, 0v_4, 1s^{\mathrm{K-K}}, 0v_3\rangle$, $|1s^{\mathrm{K-\Lambda}}, 1v_4, 1s^{\mathrm{K-K}}, 0v_3\rangle$, $|1s^{\mathrm{K-\Lambda}}, 0v_4, 2p^{\mathrm{K-K}}, 0v_3\rangle$, and $|1s^{\mathrm{K-\Lambda}}, 0v_4, 1s^{\mathrm{K-K}}, 1v_3\rangle$ is summarized in Supplementary Fig. 3b.

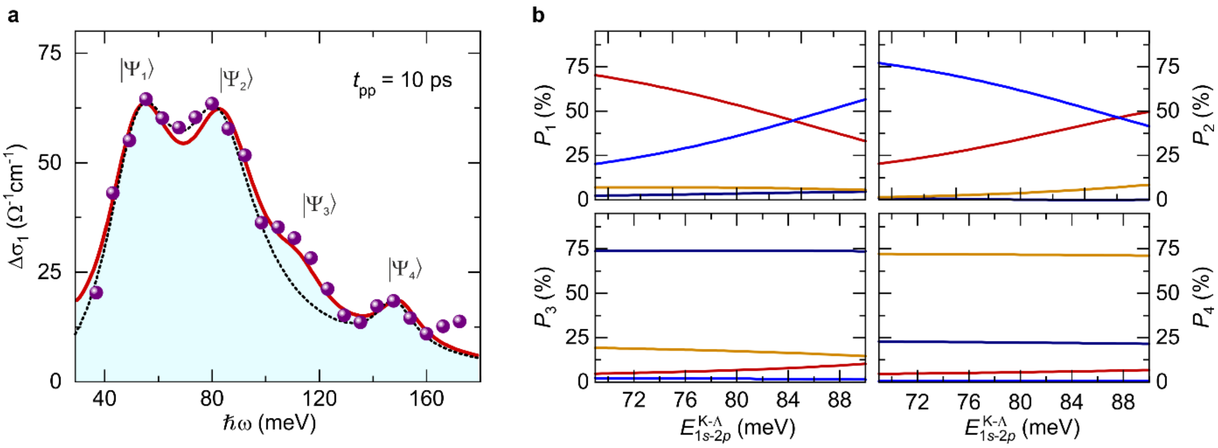

Supplementary Figure 3 | Strong mixing of oscillator strength. a, Pump-induced change of the real part of the optical conductivity $\Delta\sigma_1$ of the WSe_2 BL/gypsum heterostructure at a pump probe delay time of t_{pp} = 10 ps as a function of the photon energy. The pump fluence and the temperature were set to 23 μJcm^{-2} and 260 K, respectively. The experimental data (purple spheres) shows four absorption bands corresponding to the Lyman-polaron states $|\Psi_n\rangle$ (n = 1, 2, 3, 4). Solid lines represent the fit to the experimental data based on eq. (1). The black dashed line underpins the discrepancy arising from neglecting the coupling of K-K excitons. **b**, Projection P_{n} of the Lyman-polaron states $|\Psi_n\rangle$ (n = 1, 2, 3, 4) to the different basis sates $|2p^{\mathrm{K-\Lambda}}, 0v_4, 1s^{\mathrm{K-K}}, 0v_3\rangle$ (red line), $|1s^{\mathrm{K-\Lambda}}, 1v_4, 1s^{\mathrm{K-K}}, 0v_3\rangle$ (blue line), $|1s^{\mathrm{K-\Lambda}}, 0v_4, 2p^{\mathrm{K-K}}, 0v_3\rangle$ (navy line), $|1s^{\mathrm{K-\Lambda}}, 0v_4, 1s^{\mathrm{K-K}}, 1v_3\rangle$ (orange line) as a function of $E^{\mathrm{K-\Lambda}}_{1s-2p}$.

Supplementary Note 4

To demonstrate the effect of dielectric screening on the optical transitions of K-K and K-Λ excitons in bilayer WSe_2 we compare the dielectric response of a large-area 2H-stacked WSe_2 bilayer before and after capping it with hBN. Note that a 2H-stacked bilayer features K-K and K-Λ exciton resonances at different energies than the 3R-stacked bilayer[3]. Supplementary Figure 4 summarizes the pump-induces changes of the real part of the optical conductivity $\Delta\sigma_1$ (a) and the real part of the dielectric function $\Delta\varepsilon_1$ (b) for the bare WSe_2 bilayer (black spheres) and the hBN covered WSe_2 bilayer (blue spheres). The corresponding 1*s*-2*p* resonances for both samples are rather similar, and emerge at an energy of ~105 meV. Thus, the hBN capping layer affects the transition energy of excitons in bilayer WSe_2 to an extent that is negligible within the precision of our measurement. Such a weak effect is expected since the exciton Bohr radius (~1 nm, ref. [7-9]) is comparable to the thickness of the WSe_2 bilayer (~1.4 nm, ref. [10]). Furthermore, it has been shown that the changes of electronic and excitonic properties of monolayer TMD due to dielectric screening of the Coulomb-interaction are largest within the first 3 layers of hBN[11,12]. Hence, the K-K and K-Λ exciton transitions in a bare WSe_2 bilayer are expected to be similar to that in a hBN covered WSe_2 bilayer, as observed experimentally.

Since hBN and gypsum feature similar static dielectric constants[2,13], and the thickness is on the order of 100 nm, the bare exciton energies (without the direct exciton-phonon coupling) should not be affected by the gypsum capping layer. Therefore, the K-K and K-Λ exciton transitions in a gypsum-covered 3R-stacked bilayer should emerge at energies similar to the uncovered 3R-stacked bilayer, as stated in the manuscript.

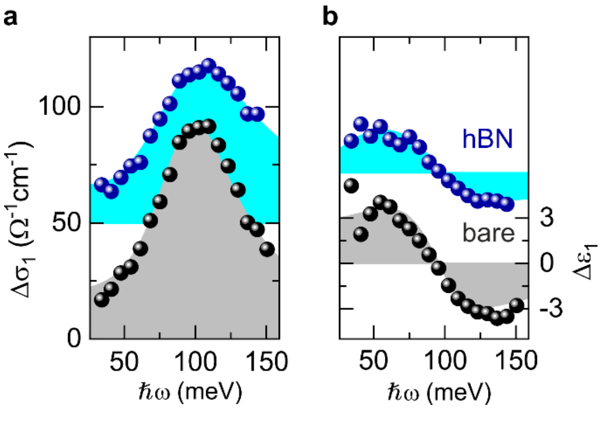

Supplementary Figure 4 | Pump-induced dielectric response of a bare and an hBN covered WSe_2 bilayer. a,**b**, Pump-induced changes of the real part of the optical conductivity $\Delta\sigma_1$ (**a**) and the dielectric function $\Delta\varepsilon_1$ (**b**) as a function of the probe photon energy for different samples at t_{pp} = 0 ps following resonant femtosecond photogeneration of 1*s* A excitons. Spheres: experimental data of the bare 2H-stacked WSe_2 bilayer (black) and the same bilayer covered with hBN (blue). The data are vertically offset for clarity. The shaded areas are fits to the experimental data based on a Lorentzian-oscillator model[3].

Supplementary Note 5

In the WSe_2/WS_2 type-II heterostructure, the intralayer excitons initially photoexcited in the WSe_2 layer undergo ultrafast charge separation at the atomic interface to form interlayer excitons within a sub-picosecond time scale. To quantitatively reproduce the experimental observations, we consider both the interlayer ($|2p^{\mathrm{inter}}, 0v_4, 1s^{\mathrm{intra}}, 0v_3\rangle$) and intralayer exciton states ($|1s^{\mathrm{inter}}, 0v_4, 2p^{\mathrm{intra}}, 0v_3\rangle$) to hybridize with manifolds of $|1s^{\mathrm{inter}}, 1v_4, 1s^{\mathrm{intra}}, 0v_3\rangle$ and $|1s^{\mathrm{inter}}, 0v_4, 1s^{\mathrm{intra}}, 1v_3\rangle$ in the gypsum layer. To account for this, we modify the Hamiltonian shown in eq. 1 in the main text by replacing $E_{1s-2p}^{\mathrm{K-\Lambda}}$ and $E_{1s-2p}^{\mathrm{K-K}}$ with $E_{1s-2p}^{\mathrm{inter}}$ and $E_{1s-2p}^{\mathrm{intra}}$, respectively. The effective Hamiltonian then reads:

$$H_{\mathrm{eff}} = \begin{pmatrix} E_{1s-2p}^{\mathrm{inter}} & V_1 & 0 & V_2 \\ V_1 & E_{\mathrm{ph}}^{v_4} & V_3 & 0 \\ 0 & V_3 & E_{1s-2p}^{\mathrm{intra}} & V_4 \\ V_2 & 0 & V_4 & E_{\mathrm{ph}}^{v_3} \end{pmatrix} \tag{S3}$$

From the numerical fitting to the experimental data, we find that the interlayer exciton-phonon interaction strength arising from direct coupling to $\mathrm{X^{intra}}$ (V_3 and V_4) is smaller than that from $\mathrm{X^{inter}}$, i.e., V_3 = 16 meV ≈ 0.7V_1 and V_4 = 25 meV ≈ 0.7V_2 (V_1 = 22 meV, V_2 = 36 meV), as shown in Supplementary Fig. 4. Here, the experimental data is reproduced satisfactorily if the reduction of V_3 and V_4 is taken into account (red line).

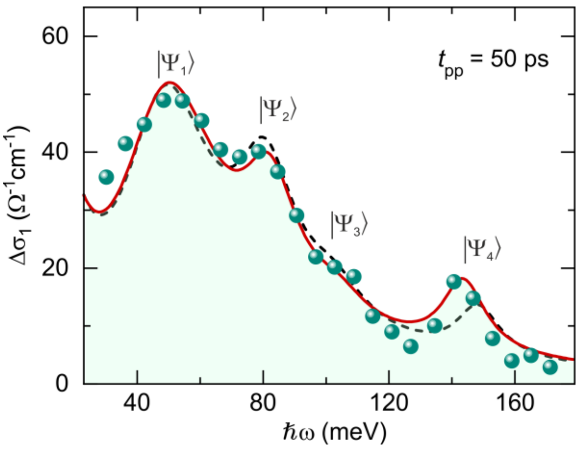

Supplementary Figure 5 | Interlayer exciton-phonon hybridization in the WSe_2/WS_2/gypsum heterostructure. Pump-induced change of the real part of the optical conductivity $\Delta\sigma_1$ of the WSe_2/WS_2/gypsum heterostructure at a pump probe delay time of t_{pp} = 50 ps as a function of the photon energy. The experimental data (green spheres) shows four absorption bands corresponding to the Lyman-polaron states $|\Psi_n\rangle$ (n = 1, 2, 3, 4). Lines represent fits to the experimental data based on eq. (S3) with V_3 = 16 meV ≈ 0.7V_1, V_4 = 25 meV ≈ 0.7V_2 (solid red line) and $V_3 = V_1$, $V_4 = V_2$ (V_1 = 22 meV, V_2 = 36 meV, dashed black line), respectively.

Supplementary Note 6

The experimental details of the ultrafast NIR pump-MIR probe spectroscopy are summarized in the Methods section. Supplementary Fig. 5 shows a schematic of the spectroscopy technique (Supplementary Fig. 5a) and the electric field of a typical MIR probe transient with its spectral amplitude and phase (Supplementary Fig. 5b, c).

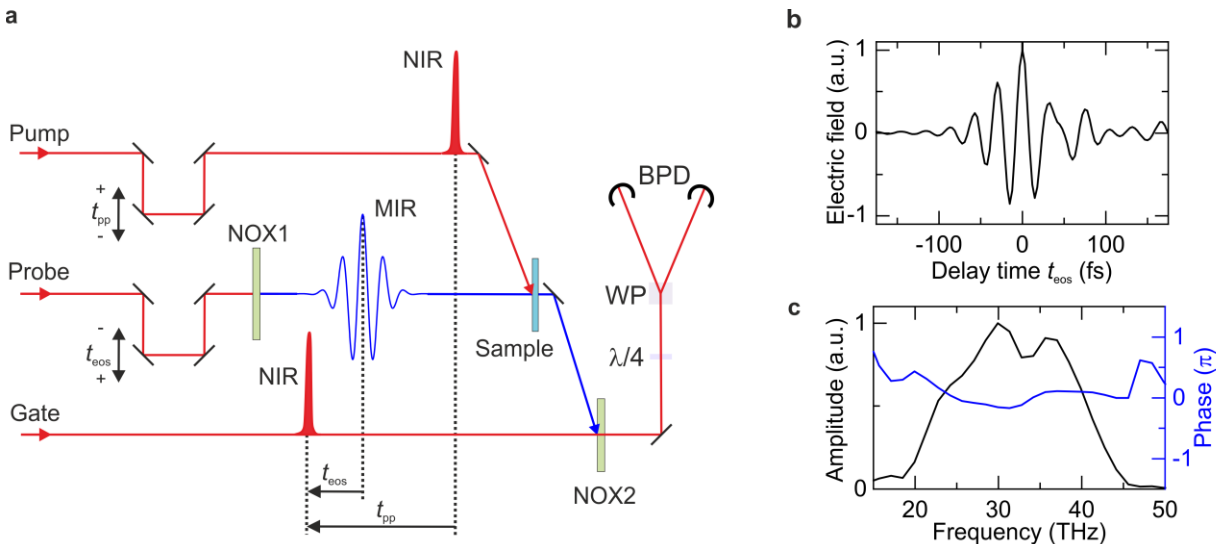

Supplementary Figure 6 | Ultrafast NIR pump-MIR probe spectroscopy. a, Schematic of the ultrafast NIR-pump-MIR probe setup. **b**, Electric field waveform of a typical MIR probe pulses as a function of the electro-optic delay time t_{eos}. **c**, Spectral amplitude and phase of the MIR probe pulses retrieved by Fourier transformation of the transient in **b** as a function of the frequency.

References

1. Steinleitner, P. *et al.* Dielectric Engineering of Electronic Correlations in a van der Waals Heterostructure. *Nano Lett.* **18**, 1402–1409 (2018).
2. Iishi, K. Phononspectroscopy and lattice dynamical calculations of anhydrite and gypsum. *Phys. Chem. Miner.* **4**, 341–359 (1979).
3. Merkl, P. *et al.* Twist-tailoring Coulomb correlations in van der Waals homobilayers. *Nat. Commun.* **11**, 2167 (2020).
4. Merkl, P. *et al.* Ultrafast transition between exciton phases in van der Waals heterostructures. *Nat. Mater.* **18**, 691–696 (2019).
5. Phillip, H. R., Taft, E. A. Kramers-Kronig Analysis of Reflectance Data for Diamond. *Phys. Rev.* **136**, A1445 (1964).
6. Dore, P. *et al.* Infrared properties of chemical-vapor deposition polycrystalline diamond windows. *Appl. Opt.* **37**, 5731-5736 (1998).
7. Chernikov, A. *et al.* Exciton Binding Energy and Nonhydrogenic Rydberg Series in Monolayer WS_2, *Phys. Rev. Lett.* **113**, 076802 (2014).
8. Poellmann, C. *et al.* Resonant internal quantum transitions and femtosecond radiative decay of excitons in monolayer WSe_2, *Nat. Mater.* **14**, 889–893 (2015).
9. Arora, A. *et al.* Interlayer excitons in a bulk van der Waals semiconductor. *Nat. Commun.* **8**, 639 (2017).
10. Tonndorf *et al.* Photoluminescence emission and Raman response of monolayer MoS_2, $MoSe_2$, and WSe_2. *Opt. Expr.* **21**, 4908–4916 (2013).
11. Li, L. H. *et al.* Dielectric Screening in Atomically Thin Boron Nitride Nanosheets. *Nano Lett.* **15**, 218–223 (2015).
12. Gerber, I. C., Marie, X. Dependence of band structure and exciton properties of encapsulated WSe_2 monolayers on the hBN-layer thickness. *Phys. Rev. B* **98**, 245126 (2018).
13. Geick, R. *et al.* Normal Modes in Hexagonal Boron Nitride. *Phys. Rev.* **146**, 543–547 (1966).
